# Mechanisms of African swine fever virus pathogenesis and immune evasion inferred from gene expression changes in infected swine macrophages

**DOI:** 10.1371/journal.pone.0223955

**Published:** 2019-11-14

**Authors:** James J. Zhu, Palaniappan Ramanathan, Elizabeth A. Bishop, Vivian O’Donnell, Douglas P. Gladue, Manuel V. Borca

**Affiliations:** 1 USDA-ARS, FADRU, Plum Island Animal Disease Center, Orient, New York, United States of America; 2 Oak Ridge Institute for Science and Education (ORISE), Oak Ridge, Tennessee, United States of America; 3 USDA-APHIS, Plum Island Animal Disease Center, Orient, New York, United States of America; Huazhong Agriculture University, CHINA

## Abstract

African swine fever (ASF) is a swine disease caused by a large, structurally complex, double-stranded DNA virus, African swine fever virus (ASFV). In domestic pigs, acute infection by highly virulent ASF viruses causes hemorrhagic fever and death. Previous work has suggested that ASFV pathogenesis is primarily mediated by host cytokines produced by infected monocytes and macrophages. To better understand molecular mechanisms mediating virus pathogenesis and immune evasion, we used transcriptome analysis to identify gene expression changes after ASFV infection in *ex vivo* swine macrophages. Our results suggest that the cytokines of TNF family including FASLG, LTA, LTB, TNF, TNFSF4, TNFSF10, TNFSF13B and TNFSF18 are the major causative cytokine factors in ASF pathogenesis via inducing apoptosis. Other up-regulated proinflammatory cytokines (IL17F and interferons) and down-regulated anti-inflammatory cytokine (IL10) may also significantly contribute to ASF pathogenesis and cause excessive tissue inflammatory responses. The differential expression of genes also indicates that ASFV could evade both the innate and adaptive immune responses by (i) inhibiting MHC Class II antigen processing and presentation, (ii) avoiding CD8+ T effector cells and neutrophil extracellular traps via decreasing expression of neutrophil/CD8+ T effector cell-recruiting chemokines, (iii) suppressing M1 activation of macrophages, (iv) inducing immune suppressive cytokines, and (v) inhibiting the processes of macrophage autophagy and apoptosis. These results provide novel information to further investigate and better understand the mechanism of pathogenesis and immune evasion of this devastating swine disease.

## Introduction

African swine fever (ASF) is an important viral disease of swine caused by African swine fever virus (ASFV), a large and structurally complex DNA virus. In domestic pigs, the clinical manifestations vary depending on the virus strains, from highly acute and lethal to subclinical. ASFV replicates mainly in macrophages and monocytes [1, 2] and induces apoptosis in the infected cells and non-infected lymphocytes in pigs [3]. TGF-β but no TNF or IL-1 was detected after *ex vivo* infection of swine macrophages and the responses to IFN-γ and LPS were suppressed in infected macrophages [4], indicating ASFV could suppress the immune response. In an *in vitro* study, TNF mRNA expression increases in ASFV-infected alveolar macrophages shortly after infection and high levels of TNF protein were detected in culture supernatants from the infected cells [5]; these culture supernatants induced apoptosis in uninfected lymphocytes, suggesting an important role of TNF in ASF pathogenesis. In an *in vivo* study, TNF and IL-1β levels increased in ASFV-infected pigs [6]. Increased prevalence of macrophages expressing TNF-α, IL-1 and IL-6 in close proximity to lymphocytes undergoing apoptosis in ASFV-infected animals has also been observed [7]. In addition, lymphopenia and neutrophilia are commonly present during ASFV infections [8, 9]. In summary, it has been frequently considered that virus pathogenesis may be mainly due to cytokines produced by infected monocytes and macrophages[10–13].

ASFV is a large, enveloped, double-stranded DNA virus with a linear genome size of 170 to 190 kbp depending on the strain, encoding between 150 and 167 open reading frames [14]. It expresses several proteins (e.g. A276R, A528R, A238, I329L, EP153R, DP71L, A224L and A179L) that have been experimentally shown *in vitro* to suppress the immune response by reducing interferon induction, interferon response and NF-κB activation [15], inhibit apoptosis of infected macrophages [16], and interfere with host gene transcription [17]. Additional research focusing on host transcription changes after infection could provide insightful information on the mechanisms of ASFV pathogenesis and immune evasion.

DNA microarrays and RNA-Seq have been used to study ASFV-infected cells. Microarrays prepared from cDNA have been used to study differences in host gene transcription between wild-type and laboratory-generated mutant ASFV *in vitro* [18, 19]. The disadvantages of these cDNA arrays are lack of full-genome coverage and gene cross-hybridization due to long probe sequences. Recently, RNA-Seq technology has been applied to the transcriptome study of pigs infected with either a highly virulent (Georgia 2007 strain) or low (OURT33) virulent ASFV [20]. The advantage of sequencing-based gene expression profiling is a probe-free approach that can provide information not included in DNA microarray; however, this approach is not cost-effective and genes with a low expression level may not show up in the data if the sequencing coverage is not deep enough. In the present study, we designed a custom whole genome expression oligo DNA microarray based on all expressed sequences aligned to the pig genome to increase gene coverage and to reduce probe redundancy, using commercial software to design probes with no or less cross-hybridization. Using these arrays, we identified genes whose expression was significantly induced or suppressed after *in vitro* infection with a highly virulent ASFV. The identified genes were further used to infer the roles of potential molecular mechanisms in ASFV pathogenesis and immune invasion.

## Methods and materials

### Cell culture of macrophages and ASFV infection

Primary swine macrophage cell cultures were derived from pig peripheral blood and were prepared as previously described [21] using an existing collection of swine blood approved IACUC protocol 205.02-17-R: Reagent Production for the Molecular Pathogenesis of Classical Swine Fever Virus (CSFV) and African Swine Fever Virus (ASFV). Macrophages were seeded in 6-well plates (Primaria Falcon, Becton Dickinson, Franklin Lakes, NY). ASFV Georgia 2007 strain [22] was used in the macrophage infection at MOI = 1. ASFV infection experiments were conducted with three biological replicates using three different animals as the source of macrophages. Mock infection was also performed in the cultured macrophages from the three commercial domestic pigs as non-infected controls.

### RNA isolation

Total RNA was extracted from primary swine macrophage cell cultures infected with the indicated virus, or mock infected at 3, 6, 9, 12, 15, and 18 hours post-infection (hpi), representing an approximate one life cycle of ASFV replication. Cells were harvested and lysed with a cell lysis buffer (Qiagen, Valencia, CA) and RNA was isolated using a RNeasy mini kit (Qiagen) according to the manufacturer’s instructions. The RNA quality was then determined using an Agilent 2100 bioanalyzer (Santa Clara, CA) using an RNA nanochip according to the procedures outlined by Agilent Technologies (Santa Clara, CA). RNA was quantified using a Nanodrop 1000 (Thermo Scientific, Waltham, MA).

### DNA microarray analysis

A 44,000 (44K) porcine whole genome expression microarray was designed based on pig expressed sequences (cDNA and EST) and porcine genome sequence homologous to non-porcine sequences. All porcine EST and RNA sequences were downloaded from the NCBI database and assembled into unique sequences using the CAP3 software program (Huang and Madan, 1999). The assembled sequences were aligned to pig genome sequences using the UCSC genome browser to select 3’ end RNA sequences or the genome sequences aligned with other expressed sequences of other species if no porcine expressed sequences were available. These selected sequences were used to design 60-mer oligonucleotide microarray probes with a low probability of cross-reacting with other genes and a bias to the 3’-end of RNA sequences using Array Designer 4.0 (Applied Biosystems, Foster City, CA). Approximately 43K porcine probes were selected to synthesize a 44K Agilent microarray for this study. The annotation of the porcine sequences was based on the results of a BLAST search against human reference proteins and RNA sequences downloaded from NCBI databases and manual curation based on all expressed sequences aligned in the porcine genome sequences using the UCSC genome browser. One hundred and eighty-six duplicated probes designed from all ASFV open reading frames were also included in this custom microarray.

The custom designed porcine microarrays were manufactured by Agilent Technologies and used for this study. Both ASFV-infected and mock-infected RNA samples were labeled with Cy3 and Cy5 individually using an Agilent low-input RNA labeling kit (Agilent Technologies). A Cy5-labeled ASFV-infected or mock-infected sample was co-hybridized with a Cy3-labeled mock-infected or ASFV-infected in one array, respectively, for each time point using a dye-swap design. The entire procedure of microarray analysis was conducted according to protocols, reagents and equipment provided or recommended by Agilent Technologies. Array slides were scanned using a GenePix 4000B scanner (Molecular Devices) with the GenePix Pro 6.0 software at 5 μM resolution.

### Statistical and bioinformatic analyses of microarray data

Background signal correction and data normalization of the microarray signals and statistical analysis were performed using the LIMMA package. Log2 fold changes in signal intensity were used in the statistical analysis to identify deferentially expressed genes. The separate-channel analysis described by Smyth and Altman (2013) was applied to calculate the p-values. To account for multiple testing, the p-values were adjusted using the Benjamini and Hochberg method and expressed as a false-discovery rate (FDR). The probe sequences were aligned to the porcine genome sequence displayed in the UCSC genome browser to validate the annotation by computational methods, such as BLAST. Gene expression differences with an FDR value of 0.05 or smaller and an expression difference ≥50% were considered statistically significant and were considered differentially expressed genes (DEG). Genes down- or up-regulated in the infected macrophages compared to the non-infected macrophages were expressed as negative and positive values (fold), respectively.

### Biological inference

The identified DEG were mapped to human reference genes. Two lists of up-regulated and down-regulated gene associated with human Entrez gene ID were analyzed with a NCBI online bioinformatics program (DAVID Bioinformatics Resources 6.8) to identify the biological pathways (GOTERM_BP_DIRECT, KEGG_PATHWAY and REACTOME_PATHWAY) significantly over-represented by DEG (FDR ≥ 0.05). The biological functions of DEG in the identified over-represented pathways associated with the immune response were based on scientific publications obtained from PubMed. Biological inferences were based on (i) the immunological functions of the DEG, (ii) gene expression levels based on microarray averaged signal intensity and (iii) magnitudes (fold) of the differential expression, assuming higher mean signal intensity and larger differentially expressed genes play a bigger biological role in the gene groups. Genes with no significantly differential expression but are known to play important roles in the biological pathways associated with the significant DEG were also used as supporting evidence. Genes down- or up-regulated in the ASFV-infected samples compared to the mock-infected samples were expressed as negative and positive values (fold), respectively. In this study, the DEG were used to infer the molecular mechanisms of ASFV pathogenesis and immune evasion.

## Results

### Host gene expression changes

**Fig 1:**
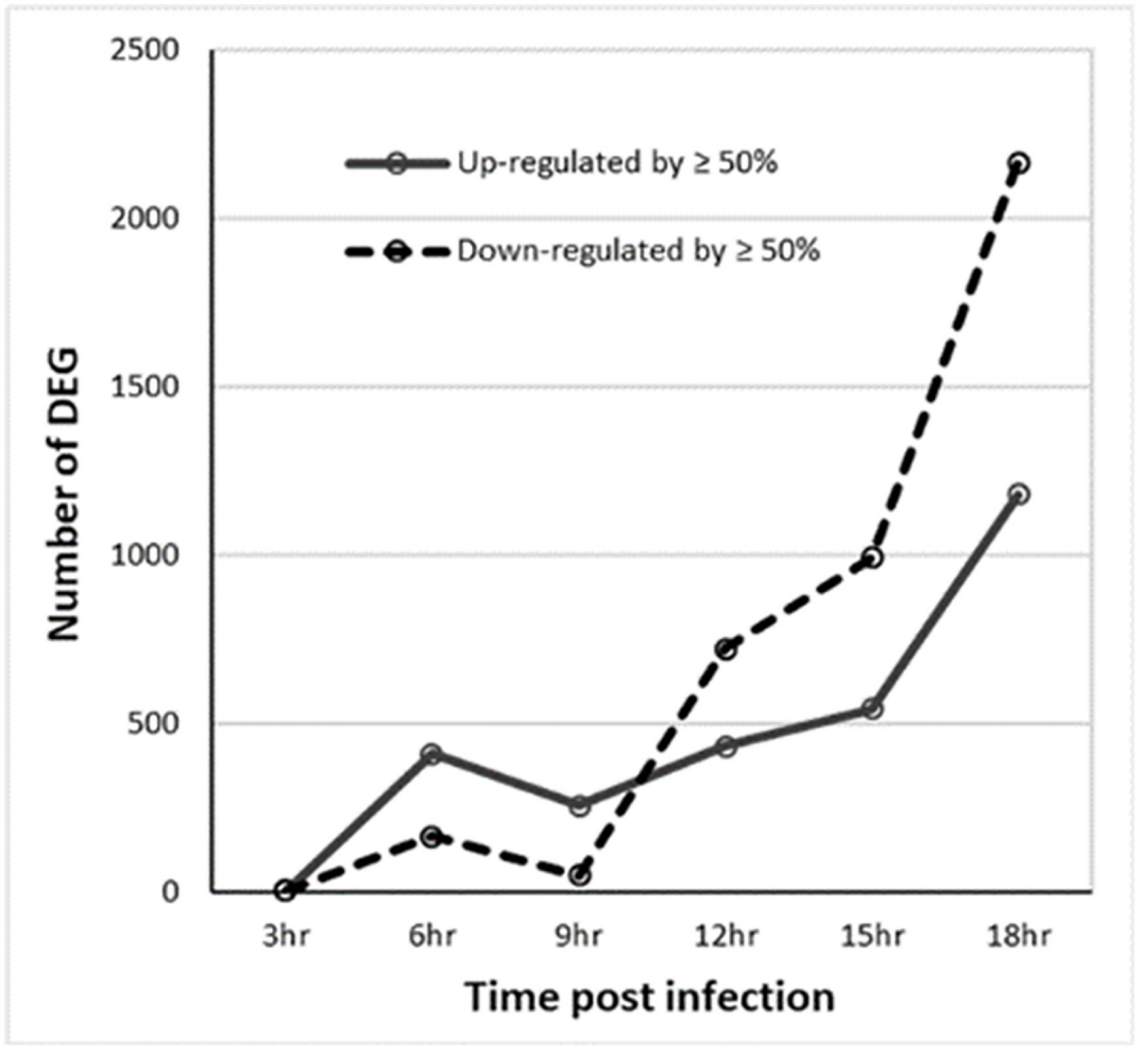
The number of significant (false discovery rate ≤0.05) differentially expressed gene (DEG) probes with a difference of 50% or great during the first 18 hours of infection.

Considering 43,264 pig gene probes, there was no significantly differential expression among the six time points evaluated in non-infected macrophages. Table 1 shows the number of probes with significantly differential expression between infected and non-infected macrophages at one or more sampling time points. Only one probe (RNA45S5) displayed significantly up-regulated expression and one (C9orf152) down-regulated expression in infected macrophages at 3 hours PI. The expression differences of most significant genes were less than 50% (Table 1). There was a total of 3,750 genes with significantly differential expression of ≥50% and significant BLAST hits with human reference genes. Fig 1 shows that the number of significantly up-regulated DEG was greater than those of down-regulated DEG at 6 and 9 hpi but smaller at 12, 15 and 18 hours. The number of differentially expressed genes decreased at 9 hpi compared to that at 6 hpi. Unlike up-regulated genes, the number of down-regulated genes displayed a significant increase at 12 hpi and the increase continued at 15 and 18 hpi.

**Table 1:** The numbers and percentages of microarray probes with significant (false discovery rate < 0.05) differential expression and significant differences by at least 50% between infected and non-infected macrophages, respectively, during the first 18 hours of ASFV infection.

Gene	3hr	6hr	9hr	12hr	15hr	18hr
Significantly up-regulated	1	2267	628	1732	1423	3315
Significantly down-regulated	1	1193	528	3075	2323	5253
Up-regulated by ≥ 50%	1	28%	59%	36%	51%	48%
Down-regulated by ≥ 50%	1	15%	11%	27%	49%	51%

### Pathway analysis

Of 3,750 DEG homologous to human genes, 1,481 genes were up-regulated and 2,340 were down-regulated. Table 2 shows the biological pathways significantly over-represented by up-regulated DEG at 6 hpi and/or later. Most of the pathways are involved in the immune response to viral infection, inflammation and stress. A large number of up-regulated genes at 6 to 18 hpi are interferon-stimulated genes (S1 Table), which agrees with up-regulated expression of all three types of interferons as shown in Table 3. The pathway results agree with the generally accepted concept of cytokine-mediated ASFV pathogenesis (Blome et al., 2013). Interestingly, twenty-four genes up-regulated at least at one time point were over-represented in tumor necrosis factor-mediated signaling pathways, indicating that TNF signaling might play a significant role in not only the immune response and but also pathogenesis. On the other hand, the biological pathways significantly over-represented by down-regulated genes are only at 15 and/or 18 hpi and some are related to the immune response, such as production of reactive nitrogen and oxygen species, autophagy, phagocytosis, and antigen processing and presentation, especially those via MHC Class II molecules (Table 4). The results of down-regulated expression would indicate a suppression of cellular machinery to favor virus replication and to evade the immune response. Therefore, a more detailed description of the differentially expressed genes is presented and discussed below.

**Table 2:** Immune-related biological pathways with over-represented genes of 1481 significantly up-regulated genes using a web tool (DAVID Bioinformatics Resources 6.8) during the first 18 hours of infection.

Pathway #	Biological pathways	Hours PI	Count^1^1	FDR^2^2
GO:0006915	apoptotic process	6	30	0.03
GO:0045824	negative regulation of innate immune response	6	6	0.00
GO:0045071	negative regulation of viral genome replication	6, 9, 12, 15	16	0.00
GO:0060333	interferon-gamma-mediated signaling pathway	6, 9, 12, 15	18	0.00
GO:0006955	immune response	6, 9, 12, 15, 18	27	0.00
GO:0009615	response to virus	6, 9, 12, 15, 18	22	0.00
GO:0032480	negative regulation of type I interferon production	6, 9, 12, 15, 18	11	0.00
GO:0045087	innate immune response	6, 9, 12, 15, 18	32	0.00
GO:0051607	defense response to virus	6, 9, 12, 15, 18	38	0.00
GO:0060337	type I interferon signaling pathway	6, 9, 12, 15, 18	25	0.00
R-HSA-877300	Interferon gamma signaling	6, 9, 12, 15, 18	21	0.00
R-HSA-909733	Interferon alpha/beta signaling	6, 9, 12, 15, 18	25	0.00
R-HSA-936440	Negative regulators of DDX58/IFIH1 signaling	6, 9, 12, 15, 18	10	0.00
GO:0006954	inflammatory response	6, 12, 18	27	0.00
GO:0032689	negative regulation of interferon-gamma production	9	6	0.04
GO:0032728	positive regulation of interferon-beta production	12	7	0.04
hsa04620	Toll-like receptor signaling pathway	12	13	0.02
hsa04622	RIG-I-like receptor signaling pathway	12	13	0.00
hsa04623	Cytosolic DNA-sensing pathway	12	12	0.00
R-HSA-918233	TRAF3-dependent IRF activation pathway	12	6	0.02
R-HSA-933541	TRAF6 mediated IRF7 activation	12	8	0.02
GO:0007165	signal transduction	12, 15, 18	52	0.00
hsa04064	NF-kappa B signaling pathway	12, 15, 18	12	0.02
GO:0002250	adaptive immune response	12, 18	16	0.00
hsa04060	Cytokine-cytokine receptor interaction	12, 18	23	0.00
R-HSA-380108	Chemokine receptors bind chemokines	12, 18	10	0.02
GO:0031295	T cell co-stimulation	15	12	0.02
GO:0050690	regulation of defense response to virus by virus	15	8	0.02
GO:0050852	T cell receptor signaling pathway	15	18	0.00
GO:0007166	cell surface receptor signaling pathway	15, 18	24	0.01
hsa04660	T cell receptor signaling pathway	15, 18	16	0.00
GO:0000184	nonsense-mediated nuclear-transcribed mRNA decay	18	23	0.00
GO:0006614	SRP-dependent co-translational protein targeting to membrane	18	20	0.01
GO:0006968	cellular defense response	18	15	0.03
GO:0007169	transmembrane receptor protein tyrosine kinase signaling pathway	18	23	0.00
GO:0019083	viral transcription	18	21	0.02
GO:0042110	T cell activation	18	15	0.00
GO:0070098	chemokine-mediated signaling pathway	18	17	0.01
GO:0072678	T cell migration	18	6	0.03
hsa04062	Chemokine signaling pathway	18	32	0.01
R-HSA-192823	Viral mRNA Translation	18	21	0.00
GO:0033209	tumor necrosis factor-mediated signaling pathway	pooled^3^	24	0.05
hsa04650	Natural killer cell mediated cytotoxicity	pooled	28	0.01
hsa05340	Primary immunodeficiency	pooled	13	0.02

^**1**^: The number of differentially expressed genes in biological process or pathways.

^**2**^: False discovery rate

^**3**^: Differentially expressed genes were pooled from all tested time points

**Table 3:** Differential expression (fold = infected/non-infected) with a FDR less than 0.05 at one or more time points and the averaged expression level (Exp) of cytokine genes between infected and non-infected macrophages at 3, 6, 9, 12, 15 and 18 hours post infection.

Gene	3hr	6hr	9hr	12hr	15hr	18hr	FDR*	Exp
IL1A	1.2	1.1	1.0	-1.1	1.2	1.0	0.55	186
IL1B_2	1.9	1.8	1.0	-1.0	1.3	1.3	0.14	975
IL1B_1	1.4	1.2	1.0	1.0	1.1	-1.0	0.21	156
IL1RN	2.0	5.5	7.4	5.8	4.6	3.9	0.00	5986
IL10	-1.1	1.2	-1.0	-2.5	-2.3	-3.1	0.00	263
IL13	1.1	1.3	1.0	1.7	2.1	2.3	0.02	45
IL16	-1.3	-1.4	-1.1	-1.5	-1.4	-1.1	0.02	975
IL17F	1.1	1.0	1.2	1.7	2.9	3.5	0.04	218
IL18BP	1.2	1.3	1.4	1.8	1.9	2.3	0.00	1023
IL27	1.8	2.8	1.8	1.6	1.4	1.3	0.01	398
CSF3	1.6	1.7	1.1	1.1	1.4	1.4	0.04	452
FASLG	1.2	1.4	1.2	1.2	1.5	1.5	0.01	84
LTA	-1.0	1.0	1.1	1.4	1.7	2.0	0.05	54
LTB	1.0	-1.0	1.1	1.8	3.0	4.1	0.01	815
TNF	1.1	1.1	1.1	1.3	1.6	1.6	0.13	213
TNFSF4	1.0	1.1	1.2	1.5	1.9	2.6	0.01	117
TNFSF10	2.4	12.8	8.7	4.5	2.4	1.7	0.00	320
TNFSF11	-1.1	-1.7	-1.3	-1.7	-1.5	-2.0	0.03	767
TNFSF13B	1.3	1.9	1.6	1.3	1.1	-1.2	0.02	1119
TNFSF15	1.0	1.2	1.1	-1.0	-1.5	-1.9	0.00	397
TNFSF18	1.5	2.2	2.1	1.7	1.3	1.1	0.01	126
IFNA	1.5	1.3	1.2	1.4	1.3	1.3	0.03	56
IFNB	3.1	1.0	1.5	3.0	3.5	3.6	0.02	79
IFNL3	1.1	1.1	1.0	1.1	1.1	1.2	0.02	49
IFNG	1.1	1.4	1.3	1.3	1.9	2.1	0.01	153

* FDR: false discovery rate. Only the smallest FDR is listed for each gene.

**Table 4:** Biological pathways with over-represented genes of 2340 significantly down-regulated genes using a web tool (DAVID Bioinformatics Resources 6.8) during the first 18 hours of infection.

Pathway #	Pathway name	Hours PI	Count	FDR
GO:0016567	protein ubiquitination	15	39	0.04
GO:0070125	mitochondrial translational elongation	15, 18	17	0.01
R-HSA-5368286	Mitochondrial translation initiation	15, 18	16	0.01
R-HSA-5389840	Mitochondrial translation elongation	15, 18	17	0.00
R-HSA-5419276	Mitochondrial translation termination	15, 18	16	0.01
GO:0002479	antigen processing and presentation of via MHC class I, TAP-dependent	18	21	0.03
GO:0006122	mitochondrial electron transport, ubiquinol to cytochrome c	18	10	0.02
GO:0006886	intracellular protein transport	18	57	0.00
GO:0006888	ER to Golgi vesicle-mediated transport	18	39	0.02
GO:0006914	autophagy	18	41	0.00
GO:0015031	protein transport	18	89	0.00
GO:0016236	macroautophagy	18	25	0.01
GO:0019886	antigen processing and presentation of via MHC class II	18	27	0.02
GO:0030433	ER-associated ubiquitin-dependent protein catabolic process	18	21	0.01
GO:0033572	transferrin transport	18	17	0.00
GO:0043161	proteasome-mediated ubiquitin-dependent protein catabolic process	18	45	0.05
GO:0045454	cell redox homeostasis	18	24	0.02
GO:0070126	mitochondrial translational termination	18	34	0.00
GO:0090383	phagosome acidification	18	13	0.03
hsa00190	Oxidative phosphorylation	18	46	0.00
hsa01100	Metabolic pathways	18	220	0.00
hsa04141	Protein processing in endoplasmic reticulum	18	52	0.00
hsa04142	Lysosome	18	45	0.00
hsa04145	Phagosome	18	51	0.00
hsa04932	Non-alcoholic fatty liver disease (NAFLD)	18	48	0.00
hsa05010	Alzheimer’s disease	18	51	0.00
hsa05012	Parkinson’s disease	18	38	0.05
R-HSA-1222556	ROS, RNS production in phagocytes	18	17	0.00
R-HSA-1236974	ER-Phagosome pathway	18	27	0.01
R-HSA-2132295	MHC class II antigen presentation	18	36	0.00
R-HSA-5628897	TP53 Regulates Metabolic Genes	18	28	0.00
R-HSA-5678895	Defective CFTR causes cystic fibrosis	18	21	0.03
R-HSA-611105	Respiratory electron transport	18	30	0.00
R-HSA-917977	Transferrin endocytosis and recycling	18	14	0.02
GO:0006886	intracellular protein transport	pooled	62	0.00
GO:0042147	retrograde transport, endosome to Golgi	pooled	23	0.03
R-HSA-1268020	Mitochondrial protein import	pooled	20	0.05
R-HSA-983168	Antigen processing: Ubiquitination & Proteasome degradation	pooled	68	0.02

### Cytokines

There are twenty-four cytokines showing significantly differential expression between infected and non-infected microphages (Table 3), twenty up-regulated and four down-regulated. IL1RN (IL-1 antagonist) and TNFSF10 (also named TNF-related apoptosis-inducing ligand or TRAIL) were the most up-regulated genes in infected macrophages by up to 7.4- and 12.8-fold, respectively, whereas IL10 was most down-regulated by up to 3.1-fold. Among twenty up-regulated, seven (FASLG, LTA, LTB, TNFSF4, TNFSF10, TNFSF13B and TNFSF18) are TNF superfamily (TNFSF) cytokines. Additionally, IL17F, an important pro-inflammatory cytokine, was expressed significantly higher at 9 hpi in infected cells and expression level increased throughout the infection. Proinflamatory cytokines IL1B and TNF which are involved in the apoptotic process werewere up-regulated by more than 60% but not at significant levels (FDR = 0.14 and 0.13, respectively). There were four genes (IL10, IL16, TNFSF11 and THFSF15) that were expressed at significantly lower levels by at least 50% compared to non-infected cells. All three types of interferons were expressed at significantly higher levels at some time points, but only IFNB and IFNG were up-regulated by more than 2-fold. CSF3 (granulocyte colony-stimulating factor) expression was significantly up-regulated at 6 hpi and up-regulated at other time points though not at significant levels. Besides IL1RN, two other immune suppressive cytokines, IL18BP (IL-18 antagonist) and IL27, were expressed significantly higher in the infected cells.

### Chemokines

There were sixteen differentially expressed chemokines at least at one-time point (Table 5). CCL4 and CXCL10 both chemoattractants for immune cells were the most up-regulated CCL and CXCL chemokines in the infected macrophages by up to 5.2- and 12.8-fold, respectively. Two CCL chemokines (CCL3 and CCL5) and two CXCL (CXCL9 and CXCL10) closely related to CCL4 and CXCL10, respectively, in sequences and functions were also expressed at higher levels in infected cells than non-infected cells. Five T helper cell-recruiting CCLs (CCL8, CCL14, CCL17, CCL20 and CCL22) were expressed at significantly higher levels at one or more time points. Among five genes, CCL8 and CCL20 expression were up-regulated only at early time points, whereas the other three were up-regulated at later time points. CCL2 and CXCL3 were the most down-regulated CCL and CXCL chemokines with up to 5.1-and 3.6-fold decreases, respectively. CXCL3 belongs to ELR+ chemokine subset that contains CXCL1, CXCL2, CXCL3, CXCL6 and CXCL8 in pigs and have aa neutrophil recruiting biological activity. Our data shows all ELR+ CXCLs were expressed with a signal intensity greater than 1000 except CXCL2 with a very low signal intensity of 77. The averaged expression of the ELR CXCLs shows down-regulation after 3 hpi. Interestingly, like ELR+ CXCLs, CXCL14, another neutrophil-recruiting chemokine, also displayed a down-regulated expression after infection.

**Table 5:** Differential expression (fold = infected/non-infected) with a FDR less than 0.05 at one or more time points and the averaged expression level (Exp) of chemokine genes between infected and non-infected macrophages at 3, 6, 9, 12, 15 and 18 hours post infection and their chemotactic activities.

Gene	3hr	6hr	9hr	12hr	15hr	18hr	FDR	Exp	Chemotactic*
CCL2	-1.0	-1.1	1.0	-1.7	-3.2	-5.1	0.01	42666	Classical Mo,NK
CCL3	1.5	1.5	-1.0	1.3	1.8	2.3	0.01	1686	NC-Mo,NK
CCL4	2.3	2.9	1.7	3.1	4.1	5.2	0.01	2433	NC-Mo,NK
CCL5	1.1	1.4	1.6	2.0	2.6	3.7	0.00	8536	NC-Mo,NK
CCL8	1.7	1.8	1.1	-1.4	-1.8	-2.6	0.01	10071	Th2
CCL14	1.0	1.1	1.0	1.3	1.5	1.8	0.01	199	?
CCL17	1.0	1.2	1.1	1.5	1.9	2.8	0.01	434	Th2
CCL20	2.4	2.3	1.4	1.2	1.2	1.1	0.00	232	Th17
CCL22	1.1	1.2	1.2	1.6	2.2	2.9	0.02	536	Th2
CXCL1	2.1	1.2	-1.2	-1.8	-1.4	-1.9	0.09	1089	N,efCD8+,M,NK
CXCL2	-1.1	-1.2	-1.2	-1.3	-1.2	-1.3	0.04	77	N,efCD8+,M,NK
CXCL3	-1.4	-2.0	-1.4	-2.6	-2.5	-3.6	0.01	1030	N,efCD8+,M,NK
CXCL6	1.8	1.0	-1.4	-1.4	-1.1	-1.9	0.12	1806	N,efCD8+,M,NK
CXCL8	1.5	1.0	-1.1	-1.4	-1.0	-1.2	0.36	1801	N,efCD8+,M,NK
CXCL9	1.3	1.5	1.3	1.5	1.5	1.2	0.09	131	CD8+,NK,Th1
CXCL10	3.7	12.8	6.5	6.4	5.9	3.5	0.00	7991	CD8+,NK,Th1
CXCL11	1.4	3.1	2.6	2.5	3.3	3.7	0.00	78	CD8+,NK,Th1
CXCL14	-1.3	-1.4	-1.1	1.0	-1.5	-1.9	0.03	1207	B,DC,N,NK,M
CXCL16	1.1	1.2	1.4	1.5	1.3	1.2	0.00	4354	CD8+,CD4+,NK
XCL1	1.2	1.6	1.7	2.0	2.5	3.2	0.01	177	DC

* The chemotactic activities are based on Griffith et al., 2014. CD8+ or CD4+: T cells; efCD8+: CD8+ effector T cell; DC: dendritic cell; N: neutrophil; NC: non-classical M: macrophage/monocyte; Mo: monocyte; NK: natural killer cells; and Th: T helper cell.

### Cell receptors

The differential expression of cell receptors critical for macrophage activation is listed in Table 6. The expression of three interferon receptors (IFNAR1, IFNAR2 and IFNGR1) were significantly down-regulated at 12, 15 and 6 hpi, respectively. Four receptors for interleukins (IL4R, IL10RA, IL10RB and IL17RA) were expressed at lower levels in infected macrophages, mostly after 9 hpi, than those in non-infected cells. IL7R is the only receptor whose expression was down-regulated at all time-points. Among differentially expressed TNF receptors, five receptors (TNFRSF1A, TNFRSF1B, TNFRSF11A, TNFRSF21 and LTBR) were down-regulated and only one up-regulated (TNFRSF25), but the down-regulated TNFRSF25 was expressed at a very low level compared to the others. These expression differences mostly occurred after 9 hours of infection. Other receptors such as three purinergic receptor (P2RX4, P2RX7 and P2RY1), two complement component 5a receptor (C3AR1 andC5AR1) and one glycolipid receptor (CD1A), which play important roles in macrophage activities, were also expressed at significantly lower levels after 9 hpi. C5AR1 expression was up-regulated at 3, 6 and 9 hpi and then decreased to become down-regulated at 15 and 18 hpi. Toll-like receptors (TLR2 and TLR4) including CD14 with average signals greater than 500 were expressed at lower levels in infected cells than those in non-infected cells after 9 hpi. The expression of two TLR receptors (TLR1 and TLR6) was also down-regulated in the infected cells. These results suggest that the sensing for TLR1, TLR2, TLR4 and TLR6 ligands in the infected cells was compromised after 9 hpi.

**Table 6:** Differential expression (fold = infected/non-infected) with a FDR less than 0.05 at one or more time points and the averaged expression level (Exp) of cell receptor genes between infected and non-infected macrophages at 3, 6, 9, 12, 15 and 18 hours post infection.

Gene	3hr	6hr	9hr	12hr	15hr	18hr	FDR	Exp
IFNAR1	-1.0	-1.2	-1.2	-1.8	-2.6	-3.6	0.00	1173
IFNAR2	1.0	1.0	1.1	-1.2	-1.6	-2.0	0.01	2210
IFNGR1	-1.3	-1.5	-1.3	-2.7	-3.4	-3.6	0.00	1073
IL10RA	-1.2	-1.1	-1.0	-2.3	-3.0	-2.9	0.00	544
IL10RB	-1.0	-1.0	1.0	-1.1	-1.3	-1.6	0.01	1970
IL17RA	-1.1	-1.3	1.0	-2.0	-2.0	-2.3	0.00	4191
IL4R	-1.3	-1.4	-1.1	-1.8	-2.0	-2.1	0.01	5753
IL7R	1.9	4.4	3.8	3.1	3.3	4.6	0.00	1853
LTBR	-1.4	-1.3	-1.1	-1.9	-3.3	-5.2	0.00	5487
TNFRSF1A	-1.3	-1.1	-1.2	-2.5	-2.9	-3.9	0.00	3846
TNFRSF1B	-1.1	-1.3	1.0	-1.2	-1.5	-1.4	0.05	11675
TNFRSF11A	1.2	1.0	-1.3	-1.8	-2.2	-2.3	0.00	251
TNFRSF25	1.1	1.1	1.2	1.6	1.8	2.2	0.02	129
TNFRSF21	1.0	-1.1	1.0	-1.3	-1.9	-3.6	0.01	8369
P2RX4	1.1	-1.0	-1.1	-1.1	-1.4	-1.6	0.04	6386
P2RX7	-1.1	-1.3	-1.3	-1.5	-2.4	-2.8	0.00	372
P2RY1	-1.7	-2.1	-1.4	-2.5	-3.4	-3.6	0.00	695
C3AR1	1.5	1.6	1.4	-1.1	-2.0	-3.5	0.02	751
C5AR1	-1.2	-1.4	-1.2	-2.5	-4.8	-7.5	0.00	13893
CD1A	1.1	1.0	-1.7	-1.5	-1.7	-1.7	0.00	749
TLR1	-1.2	-1.4	-1.1	-1.9	-2.4	-2.8	0.00	200
TLR2	-1.3	1.0	1.1	-2.2	-3.9	-5.7	0.00	10537
TLR3	1.0	1.4	1.3	1.1	-1.1	-1.1	0.02	137
TLR4	-1.2	-1.4	-1.2	-2.2	-2.9	-3.7	0.00	590
TLR5	1.0	-1.0	1.0	-1.0	1.3	1.0	0.24	41
TLR6	-1.1	-1.6	-1.5	-2.1	-2.1	-2.1	0.00	96
TLR7	1.4	2.0	1.5	-1.0	-1.1	-1.4	0.01	176
TLR8	1.1	1.1	1.0	-1.2	-1.3	-1.4	0.00	89
TLR9	-1.0	-1.1	-1.0	-1.0	-1.2	-1.2	0.00	86
TLR10	-1.0	-1.1	-1.0	-1.0	-1.0	-1.0	0.06	45
CD14	1.1	-1.1	-1.4	-1.9	-2.5	-3.4	0.01	10050

### Antigen processing and presentation genes

The expression of several genes playing roles in antigen processing and presentation to MHC class II molecules were down-regulated in the infected cells (Table 7). Cathepsins digest engulfed antigens before they can be loaded to MHC class II molecules. All highly expressed cathepsin genes were expressed at significantly lower levels by at least 2-fold in infected cells after 9 hpi when compared to non-infected cells. MHC class II DMA and DMB remove class II–associated invariant chain peptide (CLIP or CD74) from MHC class II molecules and MHC class II DOA and DOB inhibit the removal of CLIP by DMA and DMB. Interestingly, the expression of SLA-DMA and SLA-DMB were down-regulated in the infected cells mostly after 12 hpi, while the expression of SLA-DOA and SLA-DOB were up-regulated compared to non-infected cells. These results indicate that MHC class II antigen procession is compromised at 9 hpi.

**Table 7:** Differential expression (fold = infected/non-infected) with a FDR less than 0.05 at one or more time points and the averaged expression level (Exp) of genes in MHC antigen processing and presentation between infected and non-infected macrophages at 3, 6, 9, 12, 15 and 18 hours post infection.

Gene	3hr	6hr	9hr	12hr	15hr	18hr	FDR	Exp
SLA-DMB	-1.0	1.0	-1.1	-1.7	-2.7	-4.3	0.00	10386
SLA-DMA	-1.0	1.0	1.1	-1.0	-1.4	-2.1	0.02	4165
SLA-DOA	1.2	1.4	1.1	1.2	1.5	1.9	0.00	936
SLA-DOB	1.1	1.2	1.3	1.5	1.7	1.9	0.01	93
CD74 / CLIP	1.0	1.2	1.2	1.4	1.4	1.2	0.00	3546
CTSA	-1.1	-1.1	1.0	-1.1	-1.3	-1.9	0.11	34618
CTSB	-1.1	-1.2	-1.0	-1.2	-2.0	-3.8	0.04	46406
CTSC	-1.2	-1.3	-1.2	-1.4	-1.4	-1.5	0.02	1757
CTSH	-1.1	-1.1	1.1	-1.0	-1.7	-2.6	0.04	44195
CTSL	-1.1	-1.1	-1.1	-1.4	-2.1	-4.8	0.02	22078
CTSS	1.1	1.0	-1.2	-1.3	-2.4	-5.8	0.03	36684
CTSV	1.1	-1.0	-1.1	-1.2	-1.7	-2.9	0.02	6988
CALR	1.1	1.0	-1.1	-1.3	-1.5	-2.3	0.02	24056
PSMA1	1.0	1.1	1.0	-1.1	-1.4	-1.6	0.05	2436
PSMB1	-1.2	-1.2	-1.0	-1.2	-1.6	-1.9	0.02	18615
PSMB10	1.1	1.2	-1.1	-1.3	-1.4	-2.0	0.02	14974
PSMC1	-1.1	1.0	-1.0	-1.1	-1.3	-1.7	0.01	3144
PSMC3	-1.1	-1.1	-1.1	-1.2	-1.3	-1.7	0.02	14239
PSMC6	-1.2	-1.2	-1.1	-1.1	-1.2	-1.7	0.03	1433
PSMD1	-1.0	-1.3	-1.5	-1.5	-1.9	-2.3	0.00	4907
PSMD11	1.0	-1.1	-1.1	-1.1	-1.4	-1.5	0.02	915
PSMD12	-1.0	-1.1	-1.3	-1.4	-1.5	-1.6	0.01	449
PSMD2	1.1	1.0	-1.3	-1.3	-1.6	-2.0	0.01	10928
PSMD5	-1.1	-1.2	-1.1	-1.1	-1.3	-1.6	0.01	411
PSMD7	-1.2	-1.1	-1.1	-1.8	-1.9	-2.0	0.01	1101
SEL1L	-1.2	-1.3	-1.2	-1.3	-2.1	-2.7	0.00	621

### Autophagy and cell death regulating genes

There are five autophagy-related protein genes (ATG2A, ATG9A, ATG101, ATG4B and ATG7) that were significantly down-regulated in the infected cells compared to the non-infected ones (Table 8). NUPR1 (nuclear protein 1, a transcriptional regulator) inhibits autophagy-associated cell death and its expression was significantly up-regulated at all time-points by at least nearly 2-fold. BNIP3 (BCL2 interacting protein 3) is a pro-apoptosis and autophagy inducer gene, which was highly expressed in the cells tested and significantly expressed at a lower level in the infected cells after 9 hpi. These results indicated that apoptosis and autophagy were compromised in the infected cells. Another proapoptotic gene, GADD45A (growth arrest and DNA-damage-inducible 45 alpha), was also expressed at a significantly lower level in the infected cells at five time points.

**Table 8:** Differential expression (fold = infected/non-infected) with a FDR less than 0.05 at one or more time points and the averaged expression level (Exp) of autophagy and apoptosis regulating genes between infected and non-infected macrophages at 3, 6, 9, 12, 15 and 18 hours post infection.

Gene	3hr	6hr	9hr	12hr	15hr	18hr	FDR	Exp
ATG2A	-1.0	-1.2	-1.2	-1.2	-1.5	-2.0	0.01	3586
ATG9A	-1.2	-1.4	-1.2	-1.4	-1.8	-2.2	0.00	1428
ATG101	-1.3	-1.2	1.0	-1.5	-1.7	-1.7	0.01	1535
ATG4B	-1.2	-1.3	-1.2	-1.5	-1.5	-1.7	0.01	1044
ATG7	1.1	-1.1	-1.1	-1.1	-1.3	-1.6	0.01	288
NUPR1	2.0	4.3	8.3	10.8	7.3	4.3	0.00	6096
BNIP3	-1.1	-1.2	-1.4	-1.7	-1.7	-2.2	0.01	13384
GADD45A	-1.6	-1.6	-1.1	-3.5	-5.5	-6.8	0.00	804

### Signal transduction and transcription genes

Down-regulated expression of immune transcriptional factors that are important for M1 macrophage activation is listed in Table 9. CCAAT-enhancer-binding proteins (or CEBPs) is a family of transcription factors composed of six members. All six CEBP genes were expressed at significantly lower levels at two or more time points than those in non-infected macrophages. A Cbp/p300-interacting trans-activator gene, CITED4, was also expressed at a significantly lower level in the infected cells. The expression of two genes (TAB1 and TBK1) in the NFκB signaling pathway, four genes (MAP3K3, MAP3K5, MAP4K3 and MAPK7) in the MAPK-ERK pathway and four key immune transcription factors (FOS, JUN, JUND, IRF1 and IRF5) was also significantly down-regulated in the infected cells compared to non-infected cells. Two signal transducers of TNF receptors (FADD and TRADD) were also expressed at lower levels after 9 hpi. The expression of three cytokine signaling inhibitors, SIGLEC1, SOCS1, USP18, was significantly increased after ASFV infection.

**Table 9:** Differential expression (fold = infected/non-infected) with a FDR less than 0.05 at one or more time points and the averaged expression level (Exp) of immune-related transcription factor, signal transducer and signaling inhibitor genes between infected and non-infected macrophages at 3, 6, 9, 12, 15 and 18 hours post infection.

Gene	3hr	6hr	9hr	12hr	15hr	18hr	FDR	Exp
CEBPA	-1.2	-1.1	-1.1	-2.4	-3.3	-4.0	0.00	12165
CEBPB	-1.4	-1.2	-1.2	-2.7	-2.9	-3.4	0.01	3548
CEBPD	-1.5	-2.2	-1.3	-4.1	-6.5	-9.9	0.00	5493
CEBPE	-1.3	-1.2	-1.2	-1.9	-2.0	-1.8	0.01	302
CEBPG	-1.2	-1.3	-1.2	-1.5	-1.5	-1.5	0.00	258
CEBPZ	-1.2	-1.0	-1.0	-1.4	-1.7	-1.7	0.01	1504
CITED4	-1.3	-1.5	1.1	-1.8	-2.1	-2.4	0.01	4977
FOS	-1.2	-1.1	-1.1	-2.8	-2.2	-2.9	0.04	235
JUN	-1.5	-1.7	1.0	-1.9	-2.4	-2.7	0.00	734
JUND	-1.2	-1.2	-1.2	-1.7	-1.8	-1.8	0.01	2219
IRF1	1.0	1.0	-1.3	-1.8	-1.6	-1.5	0.01	1177
IRF5	1.0	1.2	1.3	-1.1	-1.4	-1.8	0.02	3662
FADD	-1.2	1.1	1.0	-1.5	-1.6	-1.6	0.01	175
MAP3K3	-1.2	-1.4	-1.0	-1.4	-1.7	-1.8	0.01	522
MAP3K5	1.0	-1.4	-1.4	-1.4	-1.4	-1.4	0.02	318
MAP4K3	-1.2	-1.2	-1.1	-1.4	-1.6	-1.8	0.00	251
MAPK7	-1.2	-1.2	-1.1	-1.2	-1.3	-1.5	0.01	1607
TAB1	-1.2	-1.4	-1.3	-1.5	-1.7	-2.1	0.00	1577
TBK1	-1.2	-1.2	-1.1	-1.7	-2.0	-2.0	0.00	897
TRADD	-1.1	-1.3	-1.3	-1.6	-2.0	-2.4	0.00	3866
SIGLEC1	1.8	5.4	6.5	6.6	6.6	4.7	0.01	1472
SOCS1	1.7	3.2	2.0	1.3	1.5	1.4	0.02	1149
USP18	1.9	5.2	5.2	5.5	4.3	3.5	0.00	5418

## Discussion

Depending on the virulence of the strains, ASFV infections cause various clinical manifestations ranging from mild clinical signs to acute hemorrhagic fever and death. Blood cell counts showed lymphopenia, monocytopenia and neutrophilia during acute and subacute infection [23]. Although the molecular mechanisms of viral hemorrhagic fevers are not clear, it is believed that a “cytokine storm” due to excessive proinflammatory cytokine responses plays an important role in the pathogenesis [11–13]. Lymphopenia, a common feature of viral hemorrhagic fever with loss of CD4+, CD8+ T cells and NK cells, is considered to be due to apoptosis mediated by pro-inflammatory cytokines and NO produced by monocytes/macrophages [24]. It was widely considered that ASF pathogenesis is mainly due to cytokines produced by infected monocytes and macrophages but the molecular basis of ASF pathogenesis is not well understood [10–13, 23] The expression of all immune cytokines has not been systemically analyzed.

In this study, we used a highly virulent isolate, ASFV Georgia strain, to infect *ex vivo* cultured macrophages and measured gene expression changes during the first 18 hours of infection in 3-hour intervals. Pathway analysis of gene expression changes indicate that ASFV infection triggered the innate immune response, immediately inducing interferon (particularly IFNβ) expression at 3 hpi and other time points (Table 3). The number of up-regulated genes increased in a linear fashion except for a drop-off at 9 hpi. On the other hand, there was a small number of down-regulated genes before 12 hpi. This number significantly increased at 12 hpi and the increase continued at 15 and 18 hpi, which coincided with significant increases in ASFV gene expression (S2 Table), indicating that the decreases in host gene expression might be due to the increase of ASFV gene expression.

Our results indicate the cytokines of the TNF family could play a major role in ASF pathogenesis based on significantly up-regulated expression of seven TNF pro-inflammatory cytokines (FASLG, LTA, LTB, TNFSF4, TNFSF10, TNFSF13B and TNFSF18) at one or more time points. Up-regulated FASLG was also detected in ASFV-infected pigs [20]. The expression of TNF was also up-regulated starting at 12 hours and continued to increase though the up-regulation was not statistically significant. These cytokines not only induce cell death/apoptosis but also cause tissue inflammation [25]. Unexpectedly, TNF expression was up-regulated but not at significant levels though the differential expression could reach the significant level at later time points. Instead, TNFSF10 or TRAIL was the most up-regulated TNF cytokine at 3 to 15 hpi. It was expressed more than TNF in this study based on the signal intensities. It was reported that FASLG and TNFSF10 can trigger apoptosis in both CD4+ and CD8+ T cells [26], which could explain the lymphopenia during ASF infection.

Neutrophilia has been reported in ASFV-infected pigs during acute and subacute infection [23]. Interestingly, the expression of CSF3/G-CSF, a neutrophil growth promoting cytokine [27], was also significantly up-regulated at 3 and 6 hpi. The up-regulation decreased at 9 and 12 hpi and then increased again at 15 and 18 hpi. Additionally, the expression of neutrophil recruiting chemokines or ELR+ CXCLs (CXCL1, 2, 3, 6 and 8) [28] were mostly downregulated after 6 hpi. These results can explain at least in part why ASFV-infected pigs develop neutrophilia.

IL17A and IL17F are two pro-inflammatory cytokines of the IL-17 family and play an important role in autoimmune and inflammatory diseases [29]. IL-17A is produced mainly in Th17 cells, whereas IL-17F was also produced in other cells [30]. In this study, IL17A was shown to be barely expressed. IL17F expression was higher than IL17A in this study (signal intensity of IL17A and IL17F: 51 and 218, respectively) and significantly up-regulated in infected macrophages after 9 hours of infection. This up-regulation could also significantly contribute to ASFV pathogenesis. Additionally, the expression of all three types of interferons was significantly induced at all time points tested. Interferons are the most potent antiviral cytokines; however, these cytokines also have other pathogenic effects such as anti-proliferation and apoptosis when they reach high concentrations [31–33]. Acute adverse effects lead to ‘flu-like’ syndromes and clinical toxicity such as myelosuppression, hemolytic anemias and thrombocytopenia could occur [34]. Therefore, based on the scale and duration of up-regulation of the interferons, we hypothesize that interferon toxicity could also play a role in ASF pathogenesis.

Unexpectedly, both IL-10 and IL10RA were expressed at significantly lower levels in the infected cells than in non-infected cells. IL-10 is a cytokine with strong anti-inflammatory activities in limiting the immune response to prevent damage to the host. The activation of mitogen-activated protein kinases (MAPKs) and nuclear factor-κB (NFκB) lead to the expression of IL-10 [35]. ASFV expresses several proteins that inhibit the signaling of these pathways [17]. We found that the expression of several genes, e.g. several highly expressed TLRs, all CEBPs, FOS, JUN, TAB1 and TBK1, in these signaling pathways was significantly down-regulated. The down-regulation of the expression coincided with the significant increase of the viral genes in our study. Because macrophage is a major source of IL-10 production during the inflammatory response [35], the down-regulation of IL-10 expression in macrophages could have a significant enhancing effect of those proinflammatory cytokines on the pathogenesis of ASF.

IL-1α, IL-1β and IL-6 expression was detected in lymphoid organs of infected animals [7]. Our results show up-regulation by less than 2-fold of IL-1β expression at 3 and 6 hpi but not at significant levels, whereas there were no changes to IL-6 expression in the infected cells (S1 Table), indicating that these cytokines probably do not play a major and/or primary role in ASF pathogenesis. Instead, the expression of IL1RN, the antagonist of IL-1 [36], was significantly up-regulated in infected macrophages at all 6 time points, supporting our conclusion. ASFV expresses a protein that can bind to IL-1β, but the deletion of this gene did not attenuate ASFV [37], which indicates ASFV possesses redundant mechanisms to interfere with IL-1 production and supports our hypothesis. Additionally, IL18BP, an antagonist of IL-18 (an important member of the IL-1 super family of cytokines) [36], was also up-regulated after infection, supporting the observation that ASFV infection suppresses IL-1 and IL-18 signaling.

IL-13 and IL-27 were two up-regulated cytokines in infected cells, which have regulatory effects on immune cells. IL-13 promotes macrophage differentiation into myeloid-derived suppressor cells that have immunosuppressive functions on various immune cells such as T, B, NK and dendritic cells [38]. IL-27 together with activation of AHR can induce the differentiation of type 1 regulatory T cells to suppress antigen-specific immune responses [39]. Interestingly, the expression of an interferon-inducible gene, IDO1 that converts tryptophan into AHR ligands [40], significantly increased after ASFV infection (S1 Table). Therefore, up-regulated expression of these cytokines in infected cells could help ASFV to evade the immune response.

Based on the differential expression of chemokines shown in Table 2 and their biological activities [28], it could be concluded that ASFV infection significantly induced expression of monocyte, T and NK cell-recruiting chemokines including CCL3, CCL4, CCL5, CXCL10 and CXCL16 but suppressed expression of neutrophil and CD8+ effector T cell chemotactic chemokines such as CXCL1, CXCL2, CXCL3, and CXCL14 after 3 hours infection. These results could also explain in part why infected pigs display neutrophilia and lymphopenia [41] and increased numbers of macrophages in infected tissues [23]. Interestingly, the expression of CCL2, a chemokine recruiting inflammatory/classical monocyte [28, 42], decreased after 9 hpi, whereas the expression of three chemokines recruiting non-classical monocytes, CCL3, CCL4 and CCL5 [42], increased mostly after ASFV infection. Neutrophils play a critical antiviral role via apoptosis-dependent phagocytosis and neutrophil extracellular traps, a networks of extracellular fibers released from neutrophils to immobilize and destroy pathogens [43]. Therefore.T, avoiding recruitment of these immune cells enhances ASFV replication and spread, whereas recruitment of other cells enhance virus spreading and TNF-induced apoptosis of T and NK cells as discussed earlier.

In a prior report, CXCL10 was the most up-regulated chemokine in infected cells, which was also found significantly up-regulated in ASFV-infected pigs [20]. Two other chemokines of this group were highly expressed after infection. Unlike other chemokines, these three chemokines can be inducedinduced by interferon. [44] and their up-regulation coincided with the increase of IFNB and IFNG expression in our study; therefore, the increased expression probably was due to the induction of interferons. CXCL10 recruits CXCR3-expressing CD8+ T and NK cells for the destruction of virus-infected cells [45] and Th1 for antigen priming [28]. In our study, ASFV infection induced interferon expression and suppressed the expression of type I and II interferon receptors after 9 hours of infection.

Immune receptors expressed on immune cells play critical roles in activation of the immune response. These receptors bind cytokines, ATP and complements released during the inflammatory response. Three interferon and five TNF receptors that bind to up-regulated cytokines were expressed at significantly lower levels in infected cells. Two critical signal transducers (TRADD and FADD) of TNF signaling pathways [46] were down-regulated in the infected cells. These results strongly suggest that ASFV-infected cells had altered gene expression to avoid activation by interferon and TNF cytokines. When cells are under stress, such as viral infection, the cells release ATP to activate the immune response via purinergic receptors, such as P2RXs [47]. The expression of three P2RX receptors was down-regulated in the infected cells. The Complement factors, C5a and C3a can activate macrophages via binding to the receptors C5AR1 and C3AR1 [48]. These two receptors were down-regulated in the infected cells. Toll-like receptors expressed on sentinel cells such as macrophages and dendritic cells recognize structurally conserved molecules derived from microbes to activate the immune response [49]. All highly expressed TLRs were also down-regulated in this study. These down-regulated receptors transmit signals to stimulate macrophage M1 activation that lead to production of nitric oxide and reactive oxygen species, important molecules in host defense against invading pathogens [50]. These results indicate that the abilities of infected macrophage to respond to M1 activation were compromised, which agrees with the observation that IFN-γ and LPS signaling were suppressed in ASFV-infected macrophages [4].

ASFV EP153R can suppress the expression of MHC class I molecules on the infected cell surface [51]. Our results indicate that the procession of MHC classes I and II antigens could be compromised based on the down-regulation of several proteases of proteasomes, endosomes and lysosomes. SLA-DMA and SLA-DMB play a critical role in epitope loading of MHC Class II molecules by removal of the invariant chain in the groove of the MHC molecules and SLA-DOA and SLA-DOB inhibit the process [52]. Our results show that DMA and DMB were down-regulated and DOA and DOB were up-regulated after 9 hours of infection, suggesting that MHC class II antigen presentation could be retarded in the infected macrophages. Additionally, TNFSF11 and TNFSF15 was expressed at lower levels after infection. TNFSF11/RANKL has been reported to play a role in enhancing DCs to stimulate naïve T cell proliferation [53], whereas TNFSF15/TL1A is expressed on antigen presenting cells and provides co-stimulatory signals to activate receptor-bearing lymphocytes [54]. Therefore, ASFV infection could not only inhibit MHC antigen presentation but also retard T cells activation by antigen presenting cells such as macrophages.

ASFV express several genes to inhibit apoptosis [16]. Our results also indicate that changes in gene expression after ASFV infection could lead to the suppression of apoptosis, especially autophagy-associated apoptosis. Apoptosis-processes were over-represented by up-regulated genes and there was significant over-representation of down-regulated genes in autophagy. NUPR1 suppresses es metabolic stress-induced autophagy-associated cell death [55]. IL-7R signaling suppresses macrophage autophagy [56]. These two genes wereere expressed at significantly higher levels in the infected cells than in non-infected cells in all time points tested. On the other hand, BNIP3 is a pro-apoptosis and autophagy inducer gene [57] and GADD45A is a proapoptotic gene [58]. Both genes were expressed at significantly lower levels after infection. Autophagy and apoptosis are well-known to play a critical role in the innate and adaptive immune response against viral infection [59, 60] including delivering endogenous antigens for MHC Class II epitope processing [61].

The molecular mechanisms involved for gene expression changes of gene expression have not yet been investigated, however, it is well-known that ASFV encodes genes such as A238L that can interfere with the host immune response [15, 62]. Several significantly down-regulated signal transducers and transcription factor genes listed in Table 9 have been shown to play an important role in M1 macrophage polarization [63, 64], these results could indicate that ASFV immune evasion could be mediated by interfering with the NFκB, JNK and IRF signaling pathways. EGRs, FOS and JUN are immediate early response genes which expression can be rapidly induced without requiring *de novo* protein synthesis during viral infection [40, 65]. Interestingly, JUN was down-regulated starting at 6 hpi and FOS was down-regulated at 12hpi, in both instances down-regulation continued into18 hpi. SIGLEC1, SOCS1 and USP18 are three cytokine signaling inhibitors [66–68], which expression was significantly increased after ASFV infection. These results further support the inhibition of M1 activation in ASFV infected macrophages.

In summary, ASFV infection significantly altered host gene profiles. Our results suggest that excessive production of proinflammatory TNF cytokines including FASLG, LTA, LTB, TNF, TNFSF4, TNFSF10, TNFSF13B, and TNFSF18 could be the major primary causative factors in ASF pathogenesis. Other up-regulated proinflammatory cytokines such as IL17F and interferons as well as down-regulated anti-inflammatory cytokines (IL10) could also significantly contribute to the pathogenesis. Our results also indicate that ASFV could evade the immune response by (i) suppressing MHC antigen processing and presentation based on down-regulated expression of cathepsins, proteasome proteases, SLA-DMA, SLA-DMB, TNFSF11 and TNFSF15 and up-regulated SLA-DOA, SLA-DOB and CD74; (ii) avoiding CD8+ cytotoxicity and neutrophil extracellular traps by decreasing expression of neutrophil/CD8+ T effector cell-recruiting chemokines; (iii) reducing M1 activation by down-regulating expression of M1-activating receptors, signal transductors and transcription factors and up-regulating expression of cytokine antagonists: IL1RN and IL18BP and cytokine IL13; (iv) inducing the expression of immune suppressive cytokines, IL-13 and IL-27, and (v) inhibiting autophagy and apoptosis via down-regulated several ATGs, BNIP3 and up-regulated NUPR1 and IL7R expression. These hypothesized mechanisms are listed in Table 10 with the references. These hypotheses and the results of this study provide insightful information for further investigation to understand this devastating swine disease.

**Table 10:** Hypothesized mechanisms of African swine fever virus pathogenesis and immune evasion inferred from differentially expressed genes (DEG) down- or up-regulated in infected macrophages with listed references used for biological inferences and tables contain the DEG.

Mechanisms	DEG	Expression	Table	Biological activity	Reference
Hemorrhagic fever by cytokine storm	TNFs, IFNs, IL17	up	4	Pro-inflammation	11–13, 29, 34
	IL10	down	4	anti-inflammation	35
Neutrophilia / inhibition of NETs^1^	CSF3	up	5	neutrophil production	27
	ELR+ CXCLs	down	5	neutrophil recruitment	28, 43
Lymphopenia / T cell suppression	TNFSF10	up	4	lymphocyte toxicity	26
	CXCL10, CXCL16	up	5	T cell recruitment	28, 45
	IL27, IDO1	up	4, S1	Treg cell differentiation	39, 40
Suppression of macrophage M1 activation	P2Rs, TLRs	down	6	macrophage M1 activation	47, 49
	C3AR1, C5AR1	down	6	macrophage M1 activation	48
	IL13	up	4	macrophage M2 activation	38
	IFN & TNF receptors	down	6	macrophage M1 activation	46, 50
Cytokine signaling inhibition	SIGLEC1, USP18, SOCS1	up	9	cytokine signaling suppression	66–68
	TRADD, FADD	down	9	TNF signal transduction	46
	IL1RN, IL18BP	up	4	IL1 & IL18 signaling inhibition	36
	12 TFs, 8 STs^2^	down	9	cytokine signal transduction	46, 63, 64
Inhibition of MHC epitope processing, loading and presentation	SLA-DMA, SLA-DMB	down	7	MHC Class II epitope loading	52
	SLA-DOA, SLA-DOB	up	7	inhibition of SLA-DM	52
	CTSs & PSMs	down	7	MHC epitope procession	52
	5 ATGs	down	8	endogenous antigen routing	61
	IL7R, NUPR1	up	6, 8	autophagy inhibition	55, 56
	GADD45, BNIP3	down	8	autophagy and apoptosis	57, 58
	TNFSF11, TNFSF15	down	4	MHC antigen presentation	53, 54
Inhibition of classical monocyte recruitment	CCL2	Down	5	classical monocytes	28, 42
	CCL3, CCL4, CCL5	up	5	non-classical monocytes	42

^1^ NETs: neutrophil extracellular traps

^2^ TFs: immune-related transcription factor and STs: signal transducers

## Supporting information

S1 Table Differential expression (fold = infected/non-infected) with a false discovery rate (FDR) less than 0.05 at one or more time points and the averaged expression level (Exp) of interferon-stimulated genes and IL6 between infected and non-infected macrophages at 3, 6, 9, 12, 15 and 18 hours post infection. (DOCX)

S2 Table The averaged microarray signal intensities of 186 ASFV open reading frame RNA at 3, 6, 9, 12, 15 and 18 hours post infection. (DOCX)

## References

[1] Malmquist WA, Hay D. Hemadsorption and cytopathic effect produced by African Swine Fever virus in swine bone marrow and buffy coat cultures. American journal of veterinary research. 1960;21:104–8. Epub 1960/01/01. . 14420403

[2] Wardley RC, Hamilton F, Wilkinson PJ. The replication of virulent and attenuated strains of African swine fever virus in porcine macrophages. Archives of virology. 1979;61(3):217–25. Epub 1979/01/01. 10.1007/bf01318056 . 496644

[3] Ramiro-Ibanez F, Ortega A, Brun A, Escribano JM, Alonso C. Apoptosis: a mechanism of cell killing and lymphoid organ impairment during acute African swine fever virus infection. The Journal of general virology. 1996;77 (Pt 9):2209–19. Epub 1996/09/01. 10.1099/0022-1317-77-9-2209 . 8811021

[4] Whittall JT, Parkhouse RM. Changes in swine macrophage phenotype after infection with African swine fever virus: cytokine production and responsiveness to interferon-gamma and lipopolysaccharide. Immunology. 1997;91(3):444–9. Epub 1997/07/01. 10.1046/j.1365-2567.1997.00272.x . 9301535 PMC1364015

[5] Gomez del Moral M, Ortuno E, Fernandez-Zapatero P, Alonso F, Alonso C, Ezquerra A, et al African swine fever virus infection induces tumor necrosis factor alpha production: implications in pathogenesis. Journal of virology. 1999;73(3):2173–80. . 9971800 10.1128/jvi.73.3.2173-2180.1999 PMC104462

[6] Salguero FJ, Ruiz-Villamor E, Bautista MJ, Sanchez-Cordon PJ, Carrasco L, Gomez-Villamandos JC. Changes in macrophages in spleen and lymph nodes during acute African swine fever: expression of cytokines. Veterinary immunology and immunopathology. 2002;90(1–2):11–22. Epub 2002/10/31. 10.1016/s0165-2427(02)00225-8 . 12406651

[7] Salguero FJ, Sanchez-Cordon PJ, Nunez A, Fernandez de Marco M, Gomez-Villamandos JC. Proinflammatory cytokines induce lymphocyte apoptosis in acute African swine fever infection. J Comp Pathol. 2005;132(4):289–302. Epub 2005/05/17. 10.1016/j.jcpa.2004.11.004 . 15893987

[8] Gomez-Villamandos JC, Bautista MJ, Carrasco L, Caballero MJ, Hervas J, Villeda CJ, et al African swine fever virus infection of bone marrow: lesions and pathogenesis. Veterinary pathology. 1997;34(2):97–107. Epub 1997/03/01. 10.1177/030098589703400202 . 9066076

[9] Wardley RC, Wilkinson PJ. The association of African swine fever virus with blood components of infected pigs. Archives of virology. 1977;55(4):327–34. Epub 1977/01/01. 10.1007/bf01315054 . 563710

[10] Blome S, Gabriel C, Beer M. Pathogenesis of African swine fever in domestic pigs and European wild boar. Virus research. 2013;173(1):122–30. Epub 2012/11/10. 10.1016/j.virusres.2012.10.026 . 23137735

[11] Teijaro JR. Cytokine storms in infectious diseases. Semin Immunopathol. 2017;39(5):501–3. Epub 2017/07/05. 10.1007/s00281-017-0640-2 . 28674818 PMC7079934

[12] Wack A, Openshaw P, O’Garra A. Contribution of cytokines to pathology and protection in virus infection. Current opinion in virology. 2011;1(3):184–95. Epub 2012/03/24. 10.1016/j.coviro.2011.05.015 . 22440716

[13] Basler CF. Molecular pathogenesis of viral hemorrhagic fever. Semin Immunopathol. 2017;39(5):551–61. Epub 2017/05/31. 10.1007/s00281-017-0637-x . 28555386 PMC6436832

[14] Dixon LK, Chapman DA, Netherton CL, Upton C. African swine fever virus replication and genomics. Virus Res. 2013;173(1):3–14. 10.1016/j.virusres.2012.10.020 . 23142553

[15] Correia S, Ventura S, Parkhouse RM. Identification and utility of innate immune system evasion mechanisms of ASFV. Virus Res. 2013;173(1):87–100. 10.1016/j.virusres.2012.10.013 . 23165138

[16] Alonso C, Galindo I, Cuesta-Geijo MA, Cabezas M, Hernaez B, Munoz-Moreno R. African swine fever virus-cell interactions: from virus entry to cell survival. Virus research. 2013;173(1):42–57. 10.1016/j.virusres.2012.12.006 . 23262167 PMC7114420

[17] Sanchez EG, Quintas A, Nogal M, Castello A, Revilla Y. African swine fever virus controls the host transcription and cellular machinery of protein synthesis. Virus research. 2013;173(1):58–75. Epub 2012/11/17. 10.1016/j.virusres.2012.10.025 . 23154157

[18] Afonso CL, Piccone ME, Zaffuto KM, Neilan J, Kutish GF, Lu Z, et al African swine fever virus multigene family 360 and 530 genes affect host interferon response. Journal of virology. 2004;78(4):1858–64. 10.1128/JVI.78.4.1858-1864.2004 . 14747550 PMC369441

[19] Zhang F, Hopwood P, Abrams CC, Downing A, Murray F, Talbot R, et al Macrophage transcriptional responses following in vitro infection with a highly virulent African swine fever virus isolate. Journal of virology. 2006;80(21):10514–21. Epub 2006/10/17. 10.1128/JVI.00485-06 . 17041222 PMC1641748

[20] Jaing C, Rowland RRR, Allen JE, Certoma A, Thissen JB, Bingham J, et al Gene expression analysis of whole blood RNA from pigs infected with low and high pathogenic African swine fever viruses. Scientific reports. 2017;7(1):10115 Epub 2017/09/02. 10.1038/s41598-017-10186-4 . 28860602 PMC5579198

[21] Zsak L, Lu Z, Kutish GF, Neilan JG, Rock DL. An African swine fever virus virulence-associated gene NL-S with similarity to the herpes simplex virus ICP34.5 gene. Journal of virology. 1996;70(12):8865–71. . 8971015 10.1128/jvi.70.12.8865-8871.1996 PMC190983

[22] Krug PW, Holinka LG, O’Donnell V, Reese B, Sanford B, Fernandez-Sainz I, et al The progressive adaptation of a georgian isolate of African swine fever virus to vero cells leads to a gradual attenuation of virulence in swine corresponding to major modifications of the viral genome. Journal of virology. 2015;89(4):2324–32. 10.1128/JVI.03250-14 . 25505073 PMC4338881

[23] Gomez-Villamandos JC, Bautista MJ, Sanchez-Cordon PJ, Carrasco L. Pathology of African swine fever: the role of monocyte-macrophage. Virus research. 2013;173(1):140–9. Epub 2013/02/05. 10.1016/j.virusres.2013.01.017 . 23376310

[24] Messaoudi I, Basler CF. Immunological features underlying viral hemorrhagic fevers. Curr Opin Immunol. 2015;36:38–46. Epub 2015/07/15. 10.1016/j.coi.2015.06.003 . 26163194 PMC4593727

[25] Croft M, Siegel RM. Beyond TNF: TNF superfamily cytokines as targets for the treatment of rheumatic diseases. Nat Rev Rheumatol. 2017;13(4):217–33. Epub 2017/03/10. 10.1038/nrrheum.2017.22 . 28275260 PMC5486401

[26] Roe MF, Bloxham DM, White DK, Ross-Russell RI, Tasker RT, O’Donnell DR. Lymphocyte apoptosis in acute respiratory syncytial virus bronchiolitis. Clin Exp Immunol. 2004;137(1):139–45. Epub 2004/06/16. 10.1111/j.1365-2249.2004.02512.x . 15196254 PMC1809083

[27] Dwivedi P, Greis KD. Granulocyte colony-stimulating factor receptor signaling in severe congenital neutropenia, chronic neutrophilic leukemia, and related malignancies. Exp Hematol. 2017;46:9–20. Epub 2016/10/30. 10.1016/j.exphem.2016.10.008 . 27789332 PMC5241233

[28] Griffith JW, Sokol CL, Luster AD. Chemokines and chemokine receptors: positioning cells for host defense and immunity. Annu Rev Immunol. 2014;32:659–702. Epub 2014/03/25. 10.1146/annurev-immunol-032713-120145 . 24655300

[29] Gu C, Wu L, Li X. IL-17 family: cytokines, receptors and signaling. Cytokine. 2013;64(2):477–85. Epub 2013/09/10. 10.1016/j.cyto.2013.07.022 . 24011563 PMC3867811

[30] Ishigame H, Kakuta S, Nagai T, Kadoki M, Nambu A, Komiyama Y, et al Differential roles of interleukin-17A and -17F in host defense against mucoepithelial bacterial infection and allergic responses. Immunity. 2009;30(1):108–19. Epub 2009/01/16. 10.1016/j.immuni.2008.11.009 . 19144317

[31] Bekisz J, Baron S, Balinsky C, Morrow A, Zoon KC. Antiproliferative Properties of Type I and Type II Interferon. Pharmaceuticals (Basel). 2010;3(4):994–1015. Epub 2010/07/29. 10.3390/ph3040994 . 20664817 PMC2907165

[32] Chawla-Sarkar M, Lindner DJ, Liu YF, Williams BR, Sen GC, Silverman RH, et al Apoptosis and interferons: role of interferon-stimulated genes as mediators of apoptosis. Apoptosis. 2003;8(3):237–49. Epub 2003/05/27. . 12766484 10.1023/a:1023668705040

[33] Apelbaum A, Yarden G, Warszawski S, Harari D, Schreiber G. Type I interferons induce apoptosis by balancing cFLIP and caspase-8 independent of death ligands. Mol Cell Biol. 2013;33(4):800–14. Epub 2012/12/12. 10.1128/MCB.01430-12 . 23230268 PMC3571350

[34] Vial T, Descotes J. Clinical toxicity of the interferons. Drug Saf. 1994;10(2):115–50. Epub 1994/02/01. 10.2165/00002018-199410020-00003 . 7516663

[35] Saraiva M, O’Garra A. The regulation of IL-10 production by immune cells. Nat Rev Immunol. 2010;10(3):170–81. Epub 2010/02/16. 10.1038/nri2711 . 20154735

[36] Mantovani A, Dinarello CA, Molgora M, Garlanda C. Interleukin-1 and Related Cytokines in the Regulation of Inflammation and Immunity. Immunity. 2019;50(4):778–95. Epub 2019/04/18. 10.1016/j.immuni.2019.03.012 . 30995499 PMC7174020

[37] Borca MV, O’Donnell V, Holinka LG, Ramirez-Medina E, Clark BA, Vuono EA, et al The L83L ORF of African swine fever virus strain Georgia encodes for a non-essential gene that interacts with the host protein IL-1beta. Virus Res. 2018;249:116–23. Epub 2018/04/02. 10.1016/j.virusres.2018.03.017 . 29605728

[38] Zhao Y, Wu T, Shao S, Shi B, Zhao Y. Phenotype, development, and biological function of myeloid-derived suppressor cells. Oncoimmunology. 2016;5(2):e1004983 Epub 2016/04/09. 10.1080/2162402X.2015.1004983 . 27057424 PMC4801459

[39] Yoshida H, Hunter CA. The immunobiology of interleukin-27. Annu Rev Immunol. 2015;33:417–43. Epub 2015/04/12. 10.1146/annurev-immunol-032414-112134 . 25861977

[40] Li Q, Harden JL, Anderson CD, Egilmez NK. Tolerogenic Phenotype of IFN-gamma-Induced IDO+ Dendritic Cells Is Maintained via an Autocrine IDO-Kynurenine/AhR-IDO Loop. J Immunol. 2016;197(3):962–70. Epub 2016/06/19. 10.4049/jimmunol.1502615 . 27316681

[41] Karalyan Z, Zakaryan H, Arzumanyan H, Sargsyan K, Voskanyan H, Hakobyan L, et al Pathology of porcine peripheral white blood cells during infection with African swine fever virus. BMC Vet Res. 2012;8:18 Epub 2012/03/01. 10.1186/1746-6148-8-18 . 22373449 PMC3308919

[42] Weber C, Belge KU, von Hundelshausen P, Draude G, Steppich B, Mack M, et al Differential chemokine receptor expression and function in human monocyte subpopulations. J Leukoc Biol. 2000;67(5):699–704. Epub 2000/05/16. 10.1002/jlb.67.5.699 . 10811011

[43] Agraz-Cibrian JM, Giraldo DM, Mary FM, Urcuqui-Inchima S. Understanding the molecular mechanisms of NETs and their role in antiviral innate immunity. Virus research. 2017;228:124–33. Epub 2016/12/08. 10.1016/j.virusres.2016.11.033 . 27923601

[44] Antonelli A, Ferrari SM, Giuggioli D, Ferrannini E, Ferri C, Fallahi P. Chemokine (C-X-C motif) ligand (CXCL)10 in autoimmune diseases. Autoimmun Rev. 2014;13(3):272–80. Epub 2013/11/06. 10.1016/j.autrev.2013.10.010 . 24189283

[45] Hickman HD, Reynoso GV, Ngudiankama BF, Cush SS, Gibbs J, Bennink JR, et al CXCR3 chemokine receptor enables local CD8(+) T cell migration for the destruction of virus-infected cells. Immunity. 2015;42(3):524–37. Epub 2015/03/15. 10.1016/j.immuni.2015.02.009 . 25769612 PMC4365427

[46] Sabio G, Davis RJ. TNF and MAP kinase signalling pathways. Semin Immunol. 2014;26(3):237–45. Epub 2014/03/22. 10.1016/j.smim.2014.02.009 . 24647229 PMC4099309

[47] Hamidzadeh K, Mosser DM. Purinergic Signaling to Terminate TLR Responses in Macrophages. Front Immunol. 2016;7:74 Epub 2016/03/15. 10.3389/fimmu.2016.00074 . 26973651 PMC4773587

[48] Bohlson SS, O’Conner SD, Hulsebus HJ, Ho MM, Fraser DA. Complement, c1q, and c1q-related molecules regulate macrophage polarization. Front Immunol. 2014;5:402 Epub 2014/09/06. 10.3389/fimmu.2014.00402 . 25191325 PMC4139736

[49] Kawasaki T, Kawai T. Toll-like receptor signaling pathways. Front Immunol. 2014;5:461 Epub 2014/10/14. 10.3389/fimmu.2014.00461 . 25309543 PMC4174766

[50] Mills CD. M1 and M2 Macrophages: Oracles of Health and Disease. Crit Rev Immunol. 2012;32(6):463–88. Epub 2013/02/23. . 23428224 10.1615/critrevimmunol.v32.i6.10

[51] Hurtado C, Bustos MJ, Granja AG, de Leon P, Sabina P, Lopez-Vinas E, et al The African swine fever virus lectin EP153R modulates the surface membrane expression of MHC class I antigens. Archives of virology. 2011;156(2):219–34. 10.1007/s00705-010-0846-2 . 21069396

[52] Blum JS, Wearsch PA, Cresswell P. Pathways of antigen processing. Annu Rev Immunol. 2013;31:443–73. Epub 2013/01/10. 10.1146/annurev-immunol-032712-095910 . 23298205 PMC4026165

[53] Anderson DM, Maraskovsky E, Billingsley WL, Dougall WC, Tometsko ME, Roux ER, et al A homologue of the TNF receptor and its ligand enhance T-cell growth and dendritic-cell function. Nature. 1997;390(6656):175–9. Epub 1997/11/21. 10.1038/36593 . 9367155

[54] Migone TS, Zhang J, Luo X, Zhuang L, Chen C, Hu B, et al TL1A is a TNF-like ligand for DR3 and TR6/DcR3 and functions as a T cell costimulator. Immunity. 2002;16(3):479–92. Epub 2002/03/26. 10.1016/s1074-7613(02)00283-2 . 11911831

[55] Hamidi T, Cano CE, Grasso D, Garcia MN, Sandi MJ, Calvo EL, et al NUPR1 works against the metabolic stress-induced autophagy-associated cell death in pancreatic cancer cells. Autophagy. 2013;9(1):95–7. Epub 2012/10/11. 10.4161/auto.22258 . 23047430 PMC3542222

[56] Zhu J, Zhang W, Zhang L, Xu L, Chen X, Zhou S, et al IL-7 suppresses macrophage autophagy and promotes liver pathology in Schistosoma japonicum-infected mice. J Cell Mol Med. 2018;22(7):3353–63. Epub 2018/03/23. 10.1111/jcmm.13610 . 29566311 PMC6010884

[57] Moriyama M, Moriyama H, Uda J, Kubo H, Nakajima Y, Goto A, et al BNIP3 upregulation via stimulation of ERK and JNK activity is required for the protection of keratinocytes from UVB-induced apoptosis. Cell Death Dis. 2017;8(2):e2576 Epub 2017/02/06. 10.1038/cddis.2017.4 . 28151469 PMC5386491

[58] Tamura RE, de Vasconcellos JF, Sarkar D, Libermann TA, Fisher PB, Zerbini LF. GADD45 proteins: central players in tumorigenesis. Current molecular medicine. 2012;12(5):634–51. Epub 2012/04/21. 10.2174/156652412800619978 . 22515981 PMC3797964

[59] Munz C. Autophagy Proteins in Viral Exocytosis and Anti-Viral Immune Responses. Viruses. 2017;9(10). Epub 2017/10/05. 10.3390/v9100288 . 28976939 PMC5691639

[60] Chen H, Ning X, Jiang Z. Caspases control antiviral innate immunity. Cell Mol Immunol. 2017;14(9):736–47. Epub 2017/07/12. 10.1038/cmi.2017.44 . 28690332 PMC5596246

[61] Munz C. Autophagy proteins in antigen processing for presentation on MHC molecules. Immunol Rev. 2016;272(1):17–27. Epub 2016/06/21. 10.1111/imr.12422 . 27319339

[62] Silk RN, Bowick GC, Abrams CC, Dixon LK. African swine fever virus A238L inhibitor of NF-kappaB and of calcineurin phosphatase is imported actively into the nucleus and exported by a CRM1-mediated pathway. The Journal of general virology. 2007;88(Pt 2):411–9. Epub 2007/01/26. 10.1099/vir.0.82358-0 . 17251557

[63] Zhou D, Huang C, Lin Z, Zhan S, Kong L, Fang C, et al Macrophage polarization and function with emphasis on the evolving roles of coordinated regulation of cellular signaling pathways. Cell Signal. 2014;26(2):192–7. Epub 2013/11/14. 10.1016/j.cellsig.2013.11.004 . 24219909

[64] Juhas U, Ryba-Stanislawowska M, Szargiej P, Mysliwska J. Different pathways of macrophage activation and polarization. Postepy Hig Med Dosw (Online). 2015;69:496–502. Epub 2015/05/20. . 25983288 10.5604/17322693.1150133

[65] Bahrami S, Drablos F. Gene regulation in the immediate-early response process. Adv Biol Regul. 2016;62:37–49. Epub 2016/05/26. 10.1016/j.jbior.2016.05.001 . 27220739

[66] Basters A, Knobeloch KP, Fritz G. USP18—a multifunctional component in the interferon response. Bioscience reports. 2018;38(6). Epub 2018/08/22. 10.1042/BSR20180250 . 30126853 PMC6240716

[67] Duncan SA, Baganizi DR, Sahu R, Singh SR, Dennis VA. SOCS Proteins as Regulators of Inflammatory Responses Induced by Bacterial Infections: A Review. Frontiers in microbiology. 2017;8:2431 Epub 2018/01/10. 10.3389/fmicb.2017.02431 . 29312162 PMC5733031

[68] Zheng Q, Hou J, Zhou Y, Yang Y, Xie B, Cao X. Siglec1 suppresses antiviral innate immune response by inducing TBK1 degradation via the ubiquitin ligase TRIM27. Cell Res. 2015;25(10):1121–36. Epub 2015/09/12. 10.1038/cr.2015.108 . 26358190 PMC4650625

