# Supplementary material for: Mechanisms of African swine fever virus pathogenesis and immune evasion inferred from gene expression changes in infected swine macrophages
Source: PLoS One. 2019 Nov 14;14(11):e0223955. doi: 10.1371/journal.pone.0223955 (PMC6855437; doi:10.1371/journal.pone.0223955)
Supplement: S1 Table — (DOCX) [file pone.0223955.s001.docx]

**Supporting Table 1**. Differential expression (fold = infected/non-infected) with a false discovery rate (FDR) less than 0.05 at one or more time points and the averaged expression level (Exp) of interferon-stimulated genes and IL6 between infected and non-infected macrophages at 3, 6, 9, 12, 15 and 18 hours post infection

| **Gene** | **3hr** | **6hr** | **9hr** | **12hr** | **15hr** | **18hr** | **FDR** | **Exp** |
| --- | --- | --- | --- | --- | --- | --- | --- | --- |
| DDX58 | 2.4 | 8.5 | 6.9 | 6.2 | 4.6 | 2.9 | 0.00 | 475 |
| HERC5 | 1.9 | 4.3 | 7.2 | 8.9 | 8.4 | 6.8 | 0.00 | 469 |
| IDO1 | 1.5 | 4.8 | 2.2 | 1.9 | 1.5 | 1.3 | 0.01 | 7998 |
| IFI27L2 | 1.6 | 2.9 | 3.6 | 5.4 | 6.4 | 7.0 | 0.00 | 1122 |
| IFI44 | 1.6 | 3.4 | 5.2 | 4.8 | 3.8 | 2.9 | 0.00 | 1169 |
| IFI44L | 1.7 | 3.4 | 4.2 | 4.3 | 5.0 | 5.3 | 0.00 | 1210 |
| IFIH1 | 1.6 | 5.7 | 3.6 | 2.7 | 2.2 | 1.9 | 0.00 | 151 |
| IFIT1 | 3.6 | 13.1 | 10.8 | 10.0 | 9.2 | 7.7 | 0.00 | 869 |
| IFIT2 | 3.4 | 10.9 | 8.1 | 7.4 | 5.5 | 4.7 | 0.00 | 911 |
| IFIT3 | 2.7 | 9.5 | 7.9 | 4.4 | 2.0 | 1.3 | 0.00 | 7917 |
| IFIT5 | 2.0 | 5.9 | 6.1 | 3.4 | 2.0 | 1.4 | 0.00 | 1087 |
| IFITM1 | 1.5 | 2.8 | 3.2 | 4.6 | 4.9 | 5.4 | 0.00 | 474 |
| IFITM3 | 1.5 | 2.6 | 3.1 | 4.1 | 5.0 | 5.7 | 0.00 | 3298 |
| ISG15 | 2.7 | 5.3 | 9.1 | 9.0 | 9.4 | 8.8 | 0.00 | 17184 |
| ISG20 | 2.1 | 8.2 | 10.9 | 13.6 | 10.6 | 9.4 | 0.00 | 1152 |
| MX1 | 2.3 | 5.9 | 13.1 | 14.6 | 14.4 | 11.8 | 0.00 | 9567 |
| MX2 | 1.9 | 4.4 | 6.6 | 6.0 | 4.8 | 3.0 | 0.00 | 5056 |
| OAS1 | 1.8 | 4.4 | 8.4 | 8.3 | 5.8 | 3.8 | 0.00 | 365 |
| RSAD2 | 2.9 | 23.9 | 25.5 | 25.6 | 17.0 | 11.5 | 0.00 | 4932 |
| IL6 | 1.0 | 1.0 | 1.0 | 1.0 | 1.0 | 1.0 | 0.76 | 43 |
