# Supplementary material for: Mechanisms of African swine fever virus pathogenesis and immune evasion inferred from gene expression changes in infected swine macrophages
Source: PLoS One. 2019 Nov 14;14(11):e0223955. doi: 10.1371/journal.pone.0223955 (PMC6855437; doi:10.1371/journal.pone.0223955)
Supplement: S2 Table — (DOCX) [file pone.0223955.s002.docx]

**Supporting Table 2**. The averaged microarray signal intensities of 186 ASFV open reading frame RNA at 3, 6, 9, 12, 15 and 18 hours post infection

| Hours post infection | 3 | 6 | 9 | 12 | 15 | 18 |
| --- | --- | --- | --- | --- | --- | --- |
| averaged signal intensity | 3914 | 6440 | 4638 | 16928 | 19090 | 22264 |
